# Feasibility and effectiveness of drop-off spots to promote walking to school

**DOI:** 10.1186/s12966-014-0136-6

**Published:** 2014-10-28

**Authors:** Griet Vanwolleghem, Sara D’Haese, Delfien Van Dyck, Ilse De Bourdeaudhuij, Greet Cardon

**Affiliations:** Department of Movement and Sport Sciences, Faculty of Medicine and Health Sciences, Ghent University, Watersportlaan 2, 9000 Ghent, Belgium; Research Foundation Flanders (FWO), Egmontstraat 5, 1000 Brussels, Belgium

**Keywords:** Active commuting to school, Primary schoolchildren, Intervention, Drop-off spots

## Abstract

**Background:**

Drop-off spots are locations in the proximity of primary schools where parents can drop off or pick up their child. From these drop-off spots children can walk to and from school. This pilot study aimed to investigate the feasibility and effectiveness of drop-off spots and to evaluate how drop-off spots are perceived by school principals, teachers and parents of 6-to-12-year old children.

**Methods:**

First, a feasibility questionnaire was completed (n = 216) to obtain parental opinions towards the implementation of drop-off spots. A drop-off spot was organized (500–800 m distance from school) in two primary schools. A within-subject design was used to compare children’s (n = 58) step counts and number of walking trips during usual conditions (baseline) and during implementation of a drop-off spot (intervention). Three-level (class-participant-condition) linear regression models were used to determine intervention effects. After the intervention, 2 school principals, 7 teachers and 44 parents filled out a process evaluation questionnaire.

**Results:**

Prior to the intervention, 96% expressed the need for adult supervision during the route to school. Positive significant intervention effects were found for step counts before/after school hours (+732 step counts/day; X^2^ = 12.2; p < 0.001) and number of walking trips to/from school (+2 trips/week; X^2^ = 52.9; p < 0.001). No intervention effect was found for total step counts/day (X^2^ = 2.0; p = 0.16). The intervention was positively perceived by the school principals and parents, but teachers expressed doubts regarding future implementation.

**Conclusion:**

This pilot study showed that implementing drop-off spots might be an effective intervention to promote children’s walking to school. Implementing drop-off spots does not require major efforts from the schools and schools can choose how and when they organize drop-off spots. However, motivating teachers and involving other volunteers (e.g. parents, grandparents) may be needed. Future studies should investigate the feasibility and effectiveness of drop-off spots in a larger sample of schools.

## Background

Engaging in walking and cycling to school is an important source of daily physical activity in 6-to-12-year olds [1-3]. Despite the numerous health benefits of active transport, many primary schoolchildren do not walk or cycle to school [4-6]. In some European countries (e.g. Belgium, the Netherlands, Denmark), the proportion of children that commutes actively to school is higher compared to other (non-) European countries (e.g. US, Australia, Spain, …) [7]. However, in Flanders (northern part of Belgium), still 47% of 6-to-12-year olds are driven to school by car [8]. Eleven to twelve year old Flemish children commute actively to school more frequently, but still 41% are driven to school by car [9].

Parental safety concerns (road safety and perceived danger from strangers) have been identified as important barriers for children’s active commuting to school [10,11]. Therefore, previous interventions promoting active transport to school mainly focused on safety issues [12,13]. The “Safe Routes to School” intervention [14] in the US aimed to provide several safe routes to school (funding the construction of safe pathways to school, providing crossing guards at major intersections, …) and to provide support for schools for traffic safety education and organization of events. Furthermore, interventions like “Walking School Bus” [15-17] and “Bicycle Train” [18] provided adult supervision (by teachers, parents, …) during the active trip to school to deal with parents’ safety concerns. These programs have a fixed route with designated “stops” and “pick up times” where children can join a supervised group to walk or cycle to school. In a systematic review of Chillón and colleagues [13], all interventions promoting active transport reported an increase in the percentage of active transport to school (ranging from 3% to 64%). Additionally, 6 of the 14 interventions reported a small effect size (Cohen’s d between 0.2 and 0.5) on active transport outcomes. Furthermore, Walking school bus interventions were effective in increasing walking to school (25%) among primary schoolchildren in the US and were positively evaluated [12,19]. Moreover, important benefits of walking school buses included strong social benefits, safety benefits and time-savings [16]. Boarnet and colleagues [20] demonstrated that the “Safe Routes to School” program was effective when the project was along the child’s usual route to school.

Besides parental safety concerns, the home-school distance has also been identified as an important predictor of children’s active commuting to school [6,9,11,21]. The home-school distance is negatively associated with active commuting to school, but it must be acknowledged that the distances, found to be feasible for active commuting to school, differ between countries and between environments. A study conducted in Australia reported that 6-to-12-year olds are more likely to commute actively to school if the home-school distance is less than 800 meters [22]. In older primary schoolchildren (11–12 years) in Flanders, D’Haese et al. [9] reported criterion distances of 1.5 km for walking and 3 km for cycling to school. In the latter study, 53% of the passive commuters to school lived further from school than the feasible active commuting distance (3 km). When developing interventions promoting active commuting to school it is important to also include those children living further away from school.

As the home-school distance is not easily modifiable, previous interventions (Walking School Bus” [15-17], “Bicycle Train” [18]) focused mainly on children living within a feasible walking or cycling distance from school [13]. In some previous walking school bus programs, children living further away could be dropped off by their parents along the route to join the walking school bus [12]. However, having to match the timing of the drop-off of the child with the timing of the walking school bus can be an important barrier for parents.

Some studies indicated that drop-off spots may offer a solution to increase the daily walking in primary schoolchildren who are usually driven to school by their parents [9,23]. A drop-off spot is a location within a feasible walking distance from the school where parents can drop off or pick up their child before or after school hours. From this spot, children can walk to or from school independently or under supervision of teachers, parents or other volunteers. Drop-off spots may be an alternative for children who cannot actively commute to school due to the large home-school distance [9,23]. Additionally, in a drop-off spot intervention, safety issues can be taken into account in order to have children walking safely to school.

To our knowledge, no studies previously evaluated drop-off spots for the promotion of walking to school among primary schoolchildren. Before implementing drop-off spots, it is important to identify possible barriers, opportunities and practical concerns towards a drop-off spot intervention. Therefore, the first aim of this pilot study was to investigate the parental opinions concerning the feasibility of drop-off spots to promote walking to school among primary schoolchildren. When developing the intervention in collaboration with the schools (specifically developed for each school), those parental opinions were taken into account. A second aim of this study was to examine the effectiveness of drop-off spots on children’s step counts and walking trips to and from school. A third aim was to study how the implemented drop-off spots were perceived by parents, teachers and school principals.

## Methods

### Participants and procedure

In Spring 2013, a convenience sample (n = 8) of primary schools in West-Flanders (northwestern part of Belgium) was contacted by phone until two primary schools agreed to participate (one located in a suburban area, 150–500 residents/km^2^ (pupils: n = 85), one located in an urban area, >500 residents/km^2^ (pupils: n = 228)).

#### Prior to the intervention: development and feasibility

Before the implementation of drop-off spots, two meetings with teachers and principals were organized in each school. The school staff was closely involved in developing the intervention to ensure that the intervention was tailored to the needs of each of the schools. During the first meeting, the protocol of the study was explained and the possibilities and barriers towards drop-off spots were discussed. In a second meeting, practical issues regarding the implementation of a drop-off spot were discussed and a specific proposal of the location, distance and organization of the drop-off spot was defined to propose to the parents. Based on the topics discussed during the school meetings, a feasibility questionnaire was developed to obtain parental opinions towards the implementation of drop-off spots. This questionnaire also included a school-specific proposal of the location of the drop-off spot. The feasibility questionnaires were given to all parents two weeks before baseline measurements and were distributed and collected through the schools. Parents of 313 primary schoolchildren (6–12 years) received a questionnaire. In total, 216 parents (69%) completed the feasibility questionnaire (suburban school (n = 56), urban school (n = 160)). Based on those parental opinions and needs from each school, the intervention (organizing a drop-off spot near each school) was developed.

#### Intervention

A within-subject design was used to study children’s step counts and number of walking trips to and from school in usual conditions (baseline) and during the implementation of a drop-off spot (intervention). In each school, one drop-off spot was implemented during one school week. In total, 141 children (6–12 years) were eligible to participate in the intervention study. Children were eligible if they used passive transport to school at least once a week, indicated by the parents in an additional question in the feasibility questionnaire. Parental informed consent to wear a pedometer during baseline and intervention was obtained from 60 parents of the 141 eligible children (response rate 43%). Both measurement periods (baseline and intervention) lasted one school week (Monday until Friday). During both weeks, children wore a pedometer and parents filled out a diary (11–12 year old children completed the diary independently [24]). There was a period of three to four weeks between baseline (April 2013) and intervention (May 2013). The weather conditions were similar in both measurement periods.

A teacher was present before the children arrived at the drop-off spot, waited for the children to arrive and walked together from the drop-off spot to school at an appointed time. Parents were asked to drop off their child during a specified time period. Parents were notified that after the appointed time, teacher supervision was no longer present. Besides the children who arrived by car, also children who already walked or cycled to school could stop at the drop-off spot and could walk together with the other children to school under supervision of a teacher. Children with a bike had to walk their bike. The organization of the drop-off spot was flexible and based on what each school indicated as feasible. Both schools organized the drop-off spot somewhat differently. In the suburban school, a drop-off spot was organized only before school hours. Parents could drop off their children between 8:15 and 8:25 AM. The drop-off spot was located in a residential area and parents could drop off their children in a cul-de-sac. In the urban school, children could use the drop-off spot before and after school hours. Before school hours, parents could drop off their children between 8:00 and 8:20 AM. Because of a high number of children from the urban school participated in the study, two departure times were organized to walk to school (a first group of children departed at 8:10 AM, the second at 8:20 AM). The urban school decided to organize the drop-off spot also after school hours because of the traffic congestion after school hours in the street of the school. Parents could pick up their child at the drop-off spot around 4:10 PM. In Belgium, primary schools run until 12:00 PM on Wednesdays. Because of practical limitations, the urban school decided not to organize the drop-off spot after school hours on Wednesday. The drop-off spot was located at a square along an approach road and was separated from the road. Prior to the intervention, a flyer with information was given in both schools to the children to hand to the parents. The information included the exact location of the drop-off spot and specific time periods when parents could drop off their children, the fact that a teacher would be present at the drop-off spot and would walk with the children to school. Parents were also informed that when they arrived later at the drop-off spot, teacher supervision was no longer present.

#### After the intervention: perception of the intervention (process evaluation)

Within one week after the intervention, a questionnaire was given to the school principals, teachers and parents to collect data on how they perceived the intervention. Parents of 119 children were eligible to fill out the process evaluation questionnaire, including parents of all children wearing pedometers and parents of children not wearing pedometers but using the drop-off spot at least once a week. In total, both school principals, nine teachers (response rate 36%) and 44 parents of the 119 eligible children (response rate 37%) filled out the process evaluation questionnaire after the intervention. The present study was approved by the Ghent University Ethics Committee (EC UZG 2013/228).

### Measurements

#### Parental feasibility questionnaire

The first section of the questionnaire contained general questions about the child (age, sex) and the parents (educational level of parents) to obtain socio-demographic information. Educational level of the parents was used as a proxy measure of children’s socio-economic status (SES). The educational level was asked for mothers and fathers separately and was based on four options: completed elementary school, completed secondary school, completed college or completed university. Children were identified as being of high SES when at least one parent reached a college or university level, or of low SES when both parents did not reach a college or university level. Secondly, parents were asked to report their child’s mode of transport to school using a question matrix [25]. In this matrix, parents filled out per season on how many days per week their child went to school using different transport modes ((1) walking, (2) cycling, (3) driven by car and (4) using public transport). In a third part, parental opinions towards the implementation of drop-off spots (feasibility) were assessed. This part consisted of 16 questions concerning general characteristics of drop-off spots (benefits, barriers, environment, use). The specific questions with the corresponding response options are outlined in Table 1.

**Table 1:** **Parental feasibility towards implementation of drop-off spots**

		**School**	
	**All (n = 216)**	**Suburban (n = 56)**	**Urban (n = 160)**
	**% agree**	**% agree**	**% agree**
***Benefits*** ^***1***^			
Implementing a drop-off spot will be beneficial to children’s health	78.9	80.0	78.5
Children will enjoy the intervention	57.5	71.4^a^	52.5
Children will have more social contact because of the intervention	89.7	92.7	88.6
I will save time when a drop-off spot will be implemented	68.3	45.5^a^	76.5
***Barriers*** ^***2***^			
Weather	58.3	64.3	56.3
Lack of time	34.7	19.6	40.0
***Environment*** ^***1***^			
Only a kiss and ride space should be available when implementing a drop-off spot	75.4	83.6	72.4
There should be green space in the surroundings of the drop-off spot	78.9	75.9	80.0
There should be no busy road in the surrounding of the drop-off spot	91.9	96.4	90.4
The drop-off spot should be separated from the road (not only on sidewalk just next to the road)	95.2	92.5	96.1
It is necessary that children do not have to cross over along the route from the drop-off spot to school	87.0	90.7	85.7
The location of the drop-off spot should be on the route to parent’s work	90.8	83.0 ^a^	93.5
***Use***			
Is adult supervision necessary when arriving at the drop-off spot?			
*Never*	0.9	0.0	1.3
*Sometimes*	13.7	14.3	13.5
*Often*	9.0	8.9	9.0
*Always*	76.4	76.8	76.3
Is adult supervision necessary during the route to school?			
*No*	4.2	8.9	2.6
*Yes, for all children*	45.8	42.9	46.8
*Yes, but only for youngest children (6-9 years)*	50.0	48.2	50.6
When (time of the day) would you use a drop-off spot?			
*Never*	6.2	9.3	5.1
*Only before school*	15.2	13.0	16.0
*Only after school*	5.7	5.6	5.8
*Before and after school*	72.9	72.2	73.1
When (time of the year) would you use a drop-off spot?			
*Never*	35.0	31.5^a^	36.2
*Entire school year*	30.0	24.1^a^	32.2
*Spring/Summer*	29.1	42.6^a^	24.2
*Autumn/Winter*	5.9	1.9^a^	7.4

^1^scored on a 5-point Likert scale ranging from totally disagree to totally agree (% of response options ‘rather agree + totally agree’ shown in table).

^2^scored yes/no (% of response option ‘yes’ shown in table).

^a^significantly different from urban school.

In a last part of the questionnaire, parental opinions towards a school-specific proposal of the organisation of the drop-off spot were asked.

#### Step counts and self-reported transport to school

Weekday step counts were objectively assessed during the baseline and intervention week using a pedometer (Omron Walking Style Pro). This pedometer has been validated to measure step counts in children [26] and it provides an hourly summary of the steps taken. Children wore a pedometer during 5 consecutive school days (Monday until Friday), at baseline and during the intervention. Children were asked to wear the pedometer during waking hours and to remove the pedometer for aquatic activities (e.g. swimming, showering) and for activities that prohibit the pedometers (e.g. contact sports).

During the intervention week, the daily number of times using the drop-off spot had to be reported in a diary, adding the reason for possible non-use. At baseline and during the intervention, parents of the 6–10 year old children were asked to report their child’s daily transport mode to school and the activities for which the pedometer was removed in the diary. The 11-to-12-year old children completed the diary independently. For every minute of reported moderate-to-vigorous physical activity for which the pedometer was removed, 150 steps were added to the daily number of step counts [27].

In total, 58 children had valid pedometer data at both baseline and intervention measurements (minimum 3 school days excluding Wednesdays) and were included in the analyses. Total step counts during the entire day and step counts before and after school hours (7:00 to 9:00 AM/4:00 PM to 5:00 PM) were calculated. On Fridays, step counts after school hours were calculated from 3:00 PM to 4:00 PM, as the primary schools end at 3:00 PM on Fridays. Walking trips to and from school were obtained through the diaries. A walking trip was identified when a child walked to or from school, also when combined with another transport mode. To calculate step counts before and after school hours and walking trips to and from school, only step counts before school hours and walking trips to school were included for children from the suburban school as the suburban school only organized a drop-off spot before school hours. Step counts before and after school hours and walking trips to and from school were included for children from the urban school, because the urban school organized a drop-off spot twice a day. Total step counts per day, step counts per day before and after school hours and weekly number of walking trips to and from school were used as main outcomes to study intervention effects.

#### Questionnaire on perception of the intervention (process evaluation)

To obtain information on how the drop-off spots were perceived, school principals, teachers and parents filled out a questionnaire. This questionnaire consisted of specific questions for school principals, teachers and parents concerning the usefulness and benefits of the drop-off spot intervention, experienced opportunities and difficulties during the intervention and future possibilities for the intervention.

The specific questions of the questionnaire for school principals, teachers and parents with the corresponding response options are outlined in Table 2.

**Table 2:** **Perception of the intervention by the school principals, teachers and parents (n = 53)**

	**School principals (n = 2)**	**Teachers (n = 7)**	**Parents (n = 44)**
	**n agree**	**n agree**	**n (% ) agree**
***Usefulness of intervention*** ^***1***^			
The intervention was well organized	---	7	35 (92.1)
It is possible to use the intervention in the future	2	2	31 (81.6)
The school has to pay more attention to safety when organizing a drop-off spot compared to the usual conditions	2	6	---
***Benefits*** ^***1***^			
The intervention gives children, who are usually dropped off by car, the opportunity to walk to school	1	6	---
Children enjoyed the intervention	1	7	31 (86.1)
Children could have more social contact with others because of this intervention	1	4	23 (65.7)
***Difficulties during intervention*** ^***2***^			
Busy traffic on the way to the drop-off spot	---	---	2 (6.8)
The time when the drop-off spot was organized did not fit	---	---	9 (20.5)
Resistance teachers	0	---	---
Resistance parents	1	1	---
Resistance children	0	0	---
Organizational limitations (e.g. willingness volunteers)	0	2	---
School environment (e.g. busy traffic in the surrounding of the school environment)	0	2	---
The intervention requires an additional load for the teachers	---	2	---
***Opportunities for intervention***			
How often would you continue to use this intervention?			
*Never*	1	3	3 (7.0)
*1*–*2 times per week*	0	3	11 (25.6)
*3*–*4 times per week*	0	0	9 (20.9)
*Every day*	1	1	20 (46.5)
When (time of the day) would you continue to use this intervention?			
*Never*	0	0	2 (4.7)
*Only before school*	1	4	19 (44.2)
*Only after school*	0	0	8 (18.6)
*Before and after school*	1	3	14 (32.6)
When (time of the year) would you continue to use this intervention?			
*Never*	0	0	2 (4.7)
*Only during theme-related periods at school*	0	3	0
*Entire school year*	1	3	20 (46.5)
*Spring/Summer*	1	1	21 (48.8)
*Autumn/Winter*	0	0	0
For which target group can this intervention be used in the future?			
*Nobody*	0	0	2 (4.7)
*Only for oldest children (10–12 years)*	0	0	3 (7.0)
*All ages*	2	7	38 (88.3)
Is adult supervision necessary during the route to school in the future?			
*No*	---	---	1 (2.4)
*Yes, for all children*	---	---	11 (26.2)
Yes, only *for the youngest children (6–9 years)*	---	---	30 (71.4)

^1^scored on a 5-point Likert scale ranging from totally disagree to totally agree (% of response options ‘rather agree + totally agree’ shown in table).

^2^scored yes/no (% of response option ‘yes’ shown in table).

### Data analysis

SPSS for Windows version 21 (SPSS Inc., Chicago, IL, USA) was used to describe the characteristics of the different samples. Descriptive statistics were used to describe the parental opinions towards the implementation of drop-off spots (feasibility) and the perception of the intervention by the school principals, teachers and parents. Additionally, chi square tests were conducted to test associations between parental opinions and the school (suburban and urban).

To determine intervention effects on total step counts/day, step counts/day before and after school hours and number of walking trips to and from school/week, three-level (class-participant-condition (i.e. baseline/intervention)) linear regression models with random intercept and fixed slope were conducted using MLwiN version 2.29. As only two primary schools were included, the clustering of participants within schools was not included as a level [28,29]. All analyses were controlled for age (continuous), sex, SES and school. When examining total step counts/day and step counts/day before and after school hours, analyses were controlled for pedometer wear time. Wednesdays were excluded from the analyses since no valid data were obtained at baseline and/or during the intervention (holiday, half day of school). The significance level was set at p < 0.05.

## Results

### Description of the samples

The characteristics of the different samples are shown in Table 3. Of the 216 children whose parents filled out the parental questionnaire before the intervention, 48.1% (n = 104) were boys. Twenty-five percent went to the suburban school (n = 56), the other 74.1% (n = 160) to the urban school. Mean age was 9.6 ± 1.7 years. In total, 11.6% of the children (n = 25) mostly cycled to school, 27.4% (n = 59) mostly walked to school, 48.4% (n = 104) were mostly dropped off by car and 12.6% (n = 27) mostly used public transport as travel mode.

**Table 3:** **Descriptive characteristics of the samples**

	**Parental feasibility sample (n = 216)**	**Intervention sample (n = 58)**
Age (years) (Mean ± SD)	9.6 ± 1.7	9.6 ± 1.7
Sex (n, %)		
*Boys*	104 (48.1)	22 (37.9)
*Girls*	112 (51.9)	36 (62.1)
School (n, %)		
*Suburban*	56 (25.9)	29 (50.0)
*Urban*	160 (74.1)	29 (50.0)
SES (n, %)		
*Low*	114 (53.0)	30 (51.7)
*High*	101 (47.0)	28 (48.3)
Transport mode to school (n, %)		
*Walking*	59 (27.4)	4 (7.0)
*Cycling*	25 (11.6)	2 (3.5)
*Driven by car*	104 (48.4)	40 (70.2)
*Public transport*	27 (12.6)	11 (19.3)

In total, 58 children had valid pedometer data at baseline and during the intervention week. This sample consisted of 22 boys (37.9%) and 36 girls (62.1%). In total, 51.7% (n = 30) had a low SES. Mean age was 9.7 ± 1.6 years. The demographic characteristics (age, sex, SES) of the subsample of children with valid pedometer data (n = 58) were comparable with those of the sample of children who dropped out (n = 83) (no consent for participation, no valid pedometer data), except that the proportion of children going to an urban school was higher for the drop out sample. In the suburban school, 56% (n = 14) of the children used the drop-off spot every day before school hours during the intervention. In the urban school, 15.4% (n = 4) used the drop-off spot every day only before school hours, 11.5% (n = 3) only after schools hours and 7.7% (n = 2) before and after school hours. Of all children, 26.5% (n = 13) never used the drop-off spot (12.0% (n = 3) in the suburban school; 38.5% (n = 10) in the urban school).

### Parental feasibility

Parental opinions (n = 216) concerning the feasibility of drop-off spots prior to the intervention are presented in Table 1. Of all parents, 89.7% agreed that there would be more social contact between children and 68.3% agreed that they would save time when a drop-off spot would be organized. Indicated barriers for using a drop-off spot were lack of time (34.7%) and weather conditions (58.3%). Of all parents, 76.4% agreed that providing adult supervision at the drop-off spot is needed and 95.8% expressed the need for adult supervision during the route to school. Of those 95.8%, 50.0% agreed that adult supervision was only needed for the youngest children (6–9 years). Regarding the environment of a drop-off spot, the majority of all parents (75.4%) agreed that only a kiss and ride zone should be provided instead of parking space. Additionally, 95.2% of all parents agreed that the drop-off spot should be separated from the road and not on the sidewalk just next to the road. About 90.8% of all parents agreed that the drop-off spot should be on the route to their work. Parental concerns regarding supervision and location of the drop-off spot were included in the development of the intervention.

### Intervention effects

Intervention effects on total step counts per day, step counts per day before and after school hours and weekly number of walking trips to and from school are described in Table 4. Positive significant intervention effects were found for step counts per day before and after school hours (+732 step counts/day; X^2^ = 12.2; p < 0.001) and number of walking trips to and from school (+2 trips/week; X^2^ = 52.9; p < 0.001). No significant intervention effect was found for total step counts per day (X^2^ = 2.0; p = 0.16).

**Table 4:** **Intervention effects on children’s step counts and number of walking trips to and from** ^**1**^ **school (n = 58)**

	**Mean total step counts per day (SD)** ^**a**^	**Mean step counts per day before and after** ^**1**^ **school hours (SD)** ^**a**^	**Mean walking trips per week**_**+**_ **to and from** ^**1**^ **school (SD)** ^**b**^
Baseline	12168 (3269)	1711 (961)	1 (2)
Intervention	11261 (3252)	2443 (1074)	3 (2)
Χ^2^	2.0	12.2***	52.9***

***p < 0.001; SD = standard deviation.

^a^analyses were controlled for: sex, age, socio-economic status, school and pedometer wear time.

^b^analyses were controlled for: sex, age, socio-economic status and school.

^1^not for children from the suburban school (suburban school only organized drop-off spot before school hours).

_+_excluding Wednesday.

Χ^2^ = chi square.

### Perception of the intervention (process evaluation)

Descriptive information of the questionnaire on how school principals (n = 2), teachers (n = 9) and parents (n = 44) perceived the intervention is shown in Table 2.

All teachers (n = 9) and 35 parents (92.1%) agreed that the drop-off spot was well organized. Both school principals and the majority of the parents (n = 31; 81.6%) agreed that drop-off spots could be used in the future, while only two teachers agreed. Concerning opportunities and future possibilities for the intervention, both school principals, seven teachers and 38 parents (88.7%) agreed to organize drop-off spots for all ages. Most parents (n = 30; 71.4%) agreed that adult supervision during the route to school is only needed for the youngest children (6–9 years). Additionally, 20 parents (46.5%) suggested organizing drop-off spots every day. Most teachers were not willing to organize drop-off spots (n = 3) or suggested to organize drop-off spots only one or two times per week (n = 3). One school principal, four teachers and 19 parents (44.2%) agreed to organize drop-off spots only before school hours. The other principal would organize drop-off spots before and after school hours. Three teachers preferred to organize drop-off spots only during theme-related periods at school (e.g. the week of active mobility), one school principal and three teachers agreed to organize drop-off spots during the entire school year. However, one school principal and 21 parents (48.8%) prefer this only in spring or summer.

During the intervention, two teachers reported organizational limitations (e.g. willingness of volunteers to supervise) and two teachers reported that the environment in the surroundings of the drop-off spot was a limitation to organize a drop-off spot (e.g. busy traffic and traffic congestions). Moreover, two teachers expressed that the intervention was an additional load for teachers. Only two parents reported busy traffic on the way to the drop-off spot (6.8%) and nine parents (20.5%) reported the time period(s) of the organized drop-off spot as an experienced difficulty.

## Discussion

The present study provided evidence that implementing drop-off spots is feasible, effective and is positively perceived by school principals and parents to promote children’s walking to school. However, teachers expressed doubts regarding future implementation.

Prior to the intervention, both schools mainly indicated organizational issues (e.g. time, location,…) regarding the implementation of drop-off spots, while parents were mainly concerned about safety issues. A requirement for parents to make use of the drop-off spot was the provision of adult supervision at the drop-off spot and during the walk to school. This was not surprising as previous studies investigating determinants of active commuting to school identified parental safety concerns (road safety and perceived danger from strangers) as main barriers for children’s active commuting to school [10,11].

Overall, we found that implementing drop-off spots in the proximity of primary schools was feasible, but that attention is required to several factors to enhance parental and teacher involvement and to ensure safety. These factors were comparable with the feasibility issues in walking school bus programs (e.g. willingness of volunteers, supervision, social benefits, time-savings) [12,19]. It is important to develop the intervention in close consultation with the schools, but some aspects of the intervention can be generalized across schools. Based on our findings, some general recommendations could be made to organize drop-off spots in the future. First, providing adult supervision is necessary in young children, but to stimulate children’s independent mobility [2,30], older primary schoolchildren (11–12 years) can walk independently to school. Secondly, the drop-off spot should be situated nearby approach roads as the majority of the parents indicated that they would use the drop-off spot if it is located on the route to their work. Third, the majority of the parents agreed that only a kiss and ride zone should be provided to drop off the children. Consequently, a drop-off spot does not necessarily have to be organized at a location with parking space, however, a zone which allows “kiss and ride” should be selected. This makes it easier for parents to drop off their children. With attention to safety, it is recommended that the drop-off spots are separated from the road (and not located on the sidewalk just next to the road). Cul-de-sacs, squares and playgrounds can be suitable locations. At these locations, children can play before they walk to school, which is beneficial for their daily physical activity levels. The feasibility study provided school-specific information to organize the drop-offs. Because a different approach is required for every school, it is important to check school and parental opinions before implementing drop-off spots and to take those school-specific opinions into account when developing the intervention.

Small but positive significant intervention effects were found for parameters regarding walking to school (steps before and after school hours; number of walking trips to and from school), demonstrating that drop-off spots are effective to promote walking to school among primary schoolchildren. The positive significant effects demonstrated that children who cannot commute actively to school (e.g. due to large home-school distance), can commute actively to school if drop-off spots are implemented in the proximity of the school. An explanation for the small effects could be the fact that the drop-off spots were not far enough from the school to induce large effects. Previous walking school bus programs reported higher increases of children’s walking [12,31]. However, in those programs larger distances were traveled (ranging from 0.5 km to 2.5 km) when children walked to school. Furthermore, D’Haese et al. [9] reported criterion distances for Flemish 11-to-12-year olds of 1.5 km for walking to school. Nevertheless, the drop-off spots in the present study were located at less than 800 m from the school in order to reach young children as well. Additionally, the location of the drop-off spot and the distance from school were chosen in cooperation with the schools and both schools did not find it feasible to increase the distance (>800 m from the school). So, increasing the distance from the drop-off spot to school may be desirable from a health promotion perspective, however, the feasibility of more distant drop-off spots remains to be demonstrated. Another explanation for the small effects could be that the days when children did not use the drop-off spots were also included in the analyses. Subsequently, the findings showed that children did not use the drop-off spot on a daily basis during the intervention week. Moreover, the intervention period lasted only one school week. Parents and children could not make a habit of their behavior. When the intervention could be implemented over a longer period and parents and children would use the drop-off spot more frequently (e.g. twice on a daily basis), effects may be larger. It is important to mention that the intervention effects were reported for a group of mainly low SES children (51.7%). It has been demonstrated that children with lower educated parents are at increased risk of negative health behaviors and outcomes [32] and that low SES parents are less likely to be reached and to participate in health promotion programs [33]. Consequently this intervention could be a promising strategy to promote walking in this at-risk group.

Additionally, we found that the intervention did not contribute to children’s total daily step counts. Possibly, the distance from the drop-off spots to the school was not large enough to contribute significantly to children’s daily step counts. Another explanation for this finding could be that children engaged in compensation behavior during the intervention. Children may have been less active during the rest of the day (e.g. less active playing during recess before the school starts) as they already walked before or after school hours due to the implementation of drop-off spots [34]. Moreover, children might have engaged in less after-school sports activities during the intervention period compared to the baseline measurements. In June (during the intervention period), community-based sports sessions in Flanders (for ball sports, gymnastics, dance,…) end (summer pause). Conversely, the baseline measurements (end April 2013- early May 2013) occurred before the sports seasons ended. This could partly explain the absence of an intervention effect on children’s daily step counts.

After the intervention, parents again reported the need to provide adult supervision during the route to school for the younger children. In general, the intervention was positively perceived by both school principals and parents. Nevertheless, the teachers expressed doubts regarding future implementation. Also the low response rate of the teachers (9 of 25 teachers filled out the questionnaire on how the intervention was perceived) demonstrates that teachers possibly experienced the intervention as an additional workload. Therefore, a possible solution could be to involve other volunteers (e.g. parents, grandparents, …) to organize and supervise the drop-off spots. This has been demonstrated to be a feasible strategy in previous walking school bus programs [12,16]. It is also of interest to note that for the organization of drop-off spots, less supervising adults are needed compared to walking school bus programs (e.g. multiple supervised routes to school), which can increase feasibility. Furthermore, our findings show that it is important to motivate teachers in order for them to be willing to include this task in their job responsibilities. However, when implementing more drop-off spots, extra volunteers and motivated teachers are needed.

Overall, the intervention aimed to increase walking to school focusing on those children living further away from school and who are usually driven to school by their parents. A major advantage of the intervention is its flexibility, as every school can implement drop-off spots that are specifically tailored to the school’s needs. When developing the intervention, the needs of the different schools and the parental opinions were taken into account, in order to create a real-life and most appropriate intervention for every school. Nevertheless, small adaptations to the intervention regarding organization (e.g. volunteers, distance) are desirable, depending on the school and its environmental context. Additionally, the intervention is free of cost and requires no large efforts from the school and the parents. Furthermore, implementing drop-off spots can be useful as part of a larger intervention to promote active transport to school in primary schools: drop-off spots can be easily implemented and commuting actively to school can become a daily habit. By implementing drop-off spots into a larger intervention (e.g. Walking School Bus, Safe Routes to School), also children not living within a feasible distance from school are reached.

The present study has some important limitations. First, it was a pilot study aiming to examine the feasibility and effectiveness of drop-off spots before implementing it into a larger-scale study. Therefore, the study involved only two schools with a small sample size, which limits power and generalizability. Also selection bias (self-selection of schools and participants; e.g. participation of most motivated parents) and the specific environment around the included schools limit generalizability. Secondly, the intervention period was rather short and no long-term effects were studied. Further research should focus on the long-term feasibility and effects of this intervention in a wider variety of primary schools. Third, the self-selection of parents to use the intervention or not could have influenced the results. In the current study, parents were free to decide whether they used the intervention or not, in contrast to other interventions at school where children do not have the choice to participate (e.g. playground interventions). Fourth, other influences on travel behavior (e.g. home location, household composition) might have influenced the results. However, it is assumed that these influences were limited since a within-subject design was used to determine the intervention effects. This study has important strengths. To our knowledge, this is the first study that implemented drop-off spots to increase children’s walking to school, specifically for those children who cannot commute actively to school because of a large home-school distance. Additionally, this is the first study that investigated both the feasibility and effectiveness of the implemented drop-off spots, and added information on how the intervention was perceived by the school and the parents. Other strengths of this study were the within-subject design, which induces high external validity, and the relatively high proportion of low SES children involved in the study. Furthermore, the use of the Omron Walking Style Pro allowed to assess steps during the entire school day and steps before and after school hours.

## Conclusions

The present pilot study showed that implementing drop-off spots might be a promising strategy to increase children’s walking to and from school and might provide an alternative for primary schoolchildren who cannot commute actively to school because of a large home-school distance. Implementing drop-off spots does not require major efforts from the schools and schools can choose how and when they organize and implement drop-off spots. Because teachers were less convinced and expressed doubts regarding future implementation, motivating teachers and involving other volunteers in the intervention may be desirable. Implementing drop-off spots may be useful as part of a larger intervention to promote active transport to school in primary schools.

## Abbreviations

km: Kilometer
m: Meter
SES: Socio-economic status
